# Molecular identification of *Trichinella* species by multiplex PCR: new insight for *Trichinella murrelli*

**DOI:** 10.1051/parasite/2017053

**Published:** 2017-12-08

**Authors:** Grégory Karadjian, Aurélie Heckmann, Giuseppe La Rosa, Edoardo Pozio, Pascal Boireau, Isabelle Vallée

**Affiliations:** 1 JRU BIPAR, ANSES, École Nationale Vétérinaire d'Alfort, INRA, OIE Collaborating Centre for Foodborne Zoonotic Parasites, Laboratory for Animal Health, 14 Rue Pierre et Marie Curie, 94701 Maisons-Alfort Cedex France; 2 European Union Reference Laboratory for Parasites, Istituto Superiore di Sanità, Viale Regina Elena 299, 00161 Rome Italy

**Keywords:** *Trichinella murrelli*, multiplex PCR, ITS1

## Abstract

In order to identify *Trichinella* at the species level, the commonly used test is a multiplex PCR, allowing the discrimination of nine out of the twelve taxa described so far. This test is based on five primer pairs amplifying fragments of the large subunit rDNA. Each taxon produces one or two bands of different sizes, resulting in a specific band pattern. By multiplex PCR, *Trichinella murrelli* shows two bands of 127 bp and 316 bp. However, a third band of 256 bp can occur. This band can lead to misidentification, since it is similar to the 253 bp band displayed by *Trichinella britovi*. BLAST analysis confirmed that the 256 bp band is from *T. murrelli*. The aim of this short note is to inform analysts that *T. murrelli* larvae may display either two- or three-band patterns.

## Abstract (fr)

Afin d'identifier les *Trichinella* au niveau de l'espèce, le test couramment utilisé est une PCR multiplex, permettant la discrimination de neuf des douze taxons décrits jusqu'à présent. Ce test est basé sur cinq paires d'amorces amplifiant des fragments de la grande sous-unité l'ADN ribosomal. Chaque taxon produit une ou deux bandes de tailles différentes, résultant en un patron de bandes spécifique. Par PCR multiplex, *Trichinella murrelli* présente deux bandes de 127 pb et 316 pb. Cependant, une troisième bande de 256 pb peut s'observer. Cette bande peut être la cause d'une erreur d'identification, car elle est similaire à la bande de 253 pb affichée par *Trichinella britovi*. L'analyse BLAST a confirmé que la bande à 256 pb provient de *T. murrelli*. Le but de cette note est d'informer les analystes que les larves de *T. murrelli* peuvent présenter des patrons à deux ou trois bandes.

## Introduction

*Trichinella* spp. are the causative agents of trichinellosis, a foodborne zoonotic disease acquired through the consumption of raw or undercooked meat infected by larvae in the muscle cells. The main sources of human infection are domestic pigs and wild boars [6,15]. Meat inspection of susceptible livestock (backyard and free-ranging pigs, horses) at slaughterhouses and game at game handling establishments is an important measure for preventing human infection [8]. On a routine basis, this inspection is internationally regulated with direct detection of larvae achieved through artificial digestion of infected muscle samples [2,3,11,16,23]. The isolation of *Trichinella* larvae from muscles of infected animals allows the removal of infected carcasses from the food chain and enables the identification of larvae at species or genotype level in order to acquire valuable epidemiological information to control these zoonotic pathogens [8,21]. To date, nine species and three genotypes have been recognized within the *Trichinella* genus [12]. Eight of these taxa have been proven to be infectious to humans, while the remaining four are considered as potentially infective to humans [21]. Species/genotypes within these taxa are morphologically indistinguishable (sibling species), and their identification relies on the use of biochemical or molecular assays [14,20,24].

The North American species *Trichinella murrelli* [17] is known to circulate freely among wild carnivore mammals in the United States [9,18,22] and Canada [7], however this zoonotic pathogen has also been documented in domestic dogs and horses [4,10,19,21]. Although *T. murrelli* has not been recorded in European wildlife, this pathogen was the causative agent of a severe human outbreak, which occurred through the consumption of raw horse-meat imported from Connecticut (USA) to France in 1985 [1,5].

The most common molecular test for *Trichinella* taxon identification is a multiplex PCR analysis, which allows unequivocal identification of nine of the 12 recognized taxa on the basis of the generation of one- or two-band patterns [20]. This test is based on the use of five primer pairs amplifying the internal transcribed spacers ITS1 and ITS2 and the expansion segment V region (ESV) of the large subunit ribosomal DNA [24]. According to this method, *T. murrelli* shows a double-band pattern of 127 bp and 316 bp.

In 2016, as part of proficiency testing to identify the species/genotype of *Trichinella* larvae, the National Reference Laboratories (NRLs) for Parasites in European Union member states reported a three-band pattern for *T. murrelli* larvae instead of the expected two-band pattern [20,24]. The aim of this work was to investigate whether the extra band belongs to *T. murrelli* or is a faint band caused by slightly modified protocols (Table 1).

**Table 1:** Multiplex PCR fragment sizes of the 12 taxa of the genus *Trichinella*.

Primer set	Locus^a^	*Trichinella* taxon fragment size (bp)										
			Tsp	Tna/Tpat	Tbr/T8/T9	Tps	Tmu	T6	Tne	Tpa	Tzi	
I	ESV		173	127	127	310–360	127	127	155	240	264	
II	ITS1				253		**256**					
III	ITS1							210				
IV	ITS2						316					
V	ITS2								404			

*Trichinella spiralis*(Tsp), *T. nativa*(Tna), *T. britovi*(Tbr), *T. pseudospiralis*(Tps), *T. murrelli*(Tmu), *T. nelsoni*(Tne), *T. papuae*(Tpa), *T. zimbabwenzis*(Tzi), *T. patagoniensis*(Tpat) and *Trichinella genotypes*T6, T8 and T9. The size (bp) of the ITS1 fragment of *T. murrelli* is in bold. The fragment sizes are those from Zarlenga et al. (1999) [24], Pozio and La Rosa (2010) [20] and Krivokapich et al. (2012) [13].

a ESV, expansion segment V region of the ribosomal DNA repeat; ITS1 and ITS2, internal transcribed spacers 1 and 2.

## Materials and methods

### *Trichinella* larvae

Muscle larvae were collected from CD1 or OF-1 female mice infected by four *T. murrelli* isolates (codes ISS35, ISS246, ISS346, and ISS415; www.iss.it/Trichinella/), and by a *T. britovi* isolate (code ISS100) by HCl-pepsin digestion, according to a published protocol [3].

### DNA isolation

The DNA was extracted using the DNA IQ System Kit (PROMEGA, DC6701) and the Tissue and Hair Extraction Kit (PROMEGA, DC6740) with a few modifications. Briefly, 20 μL of incubation buffer with DTT and proteinase K were added to larvae and incubated at 55 °C for 30 min shaking at 1,400 vibrations per min. Then, 40 μL of lysis buffer with DTT and 4 μL of paramagnetic resin were added. The entire solution was incubated at 25 °C for 5 min in a thermoblock without vibration, with a single vortexing step performed at mid time. Tubes were then placed in a magnetic separation stand for 1 min. The liquid phase was discarded. Then 100 μL of lysis buffer were added and resin particles were re-suspended before tubes were replaced on a paramagnetic stand and the liquid phase removed. The samples were washed four times using 100 μL washing buffer. The particles were then air-dried for 15 min and samples were eluted using 50 °μL of elution buffer for 5 min at 65 °C shaking at 1,400 vibrations per min.

### Multiplex and uniplex PCR

Five primer pairs were used in a multiplex PCR as described by Zarlenga *et al.* (1999) [24] (Primer set I, ESV target locus, 5′-GTTCCATGTGAACAGCAGT-3′, 5′-CGAAAACATACGACAACTGC-3′; primer set II, ITS1 target locus, 5′-GCTACATCCTTTTGATCTGTT-3′, 5′-AGACACAATA TCAACCACAGTACA-3′; primer set III, ITS1 target locus 5′-GCGGAAGGATCATTATCGTGTA-3′, 5′-TGGATTACAAAGAAAACCATCACT-3′; primer set IV, ITS2 target locus 5′-GTGAGCGTAATAAAGGTGCAG-3′, 5′-TTCATCACACATCTTCCACTA-3′; and primer set V, ITS2 target locus 5′-CAATTGAAAACCGCTTAGCGTGTTT-3′, 5′-TGATCTGAGGTCGACATTTCC-3′. Reactions were performed in 15 μL of 2X GoTaq^®^ Hot Start Green MasterMix (PROMEGA, M5122), 9 μL of nuclease free water, 1 μL of total primers, and 5 μL of extracted DNA.

The uniplex PCR was performed using the same mix as above but with only primer set II for the ITS1 locus at a final concentration of 10 °μM.

The PCR cycles for both multiplex and uniplex PCR were performed as follows: a pre-denaturation and polymerase activation step at 95 °C for 2 min, then 35 amplification cycles (denaturation at 95 °C for 10 sec, hybridization at 55 °C for 30 sec, and elongation at 72 °C for 30 sec), and a final elongation step at 72 °C for 5 min.

### Electrophoresis and sequencing

Agarose (Ozyme, LON50004) gels (2%) were prepared in TAE (2M Tris-acetate, 50 mM EDTA, pH 8.3) (Lonza, BE51216) solution with 5 ng/mL of ethidium bromide (Sigma, E1510). Electrophoresis was performed using 10 μL of PCR products with a 50 bp O'Range Ruler DNA ladder (Fermentas, SM0613) for 30 min at 100 V. PCR products were sequenced using the appropriate primers by Eurofins-MWG (Plateforme de l'Hôpital Cochin, Paris, France).

## Results and Discussion

**Figure 1:**
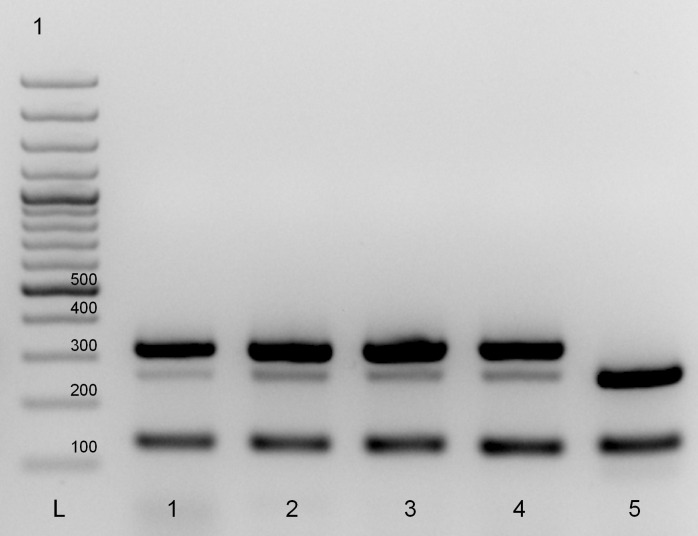
Electrophoretic profiles of *Trichinella* *murrelli* and *T. britovi* larva amplicons after multiplex PCR amplification.DNA extracts from 1 and 10 larvae of *T. murrelli* (isolate code ISS35) in lane 1 and lanes 2–4, respectively; and of *T. britovi* (isolate code ISS235) larva in lane 5. Lane L1 = 100 bp ladder.

Following multiplex PCR amplification, *T. murrelli* larvae displayed two- or three-band patterns independently of the isolate and the laboratory where the test was performed. A three-band pattern of 127 bp, 256 bp and 316 bp was observed by the French NRL (Figure 1), whereas a two-band pattern (127 bp and 316 bp) or a three-band pattern (127 bp, 256 bp and 316 bp) were found by the European Union Reference Laboratory for Parasites (EURLP) in Rome. Using the same multiplex PCR analysis protocol, *T. britovi* larvae displayed the expected band pattern of 127 bp and 253 bp (Figure 1).

**Figure 2:**
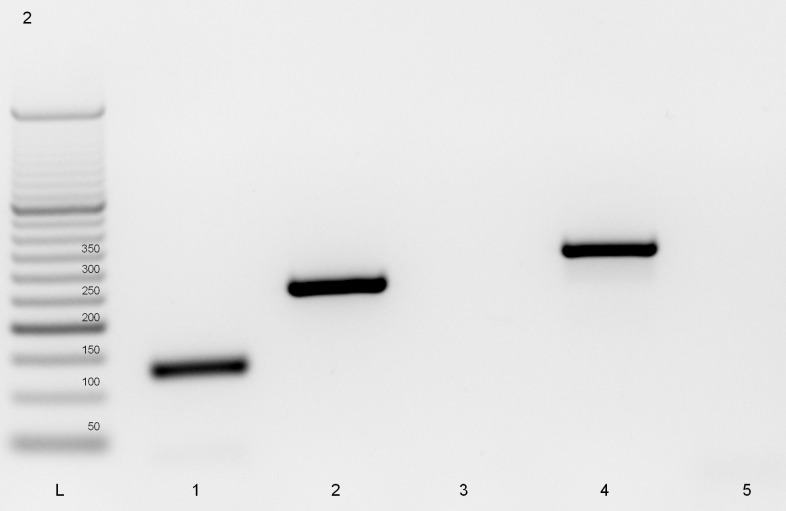
Electrophoretic profiles of *Trichinella* *murrelli* uniplex PCR amplifications.DNA from *T. murrelli* (isolate code ISS35) reference larvae was used. Lane L = 50 bp ladder. The genes targeted were the Expansion Segment V (ESV, lane 1), Internal Transcribed Spacer 1 II (ITS1 II, Lane 2), ITS1 III (lane 3), ITS2 IV (lane 4), and ITS2 V (lane 5).

**Figure 3:**
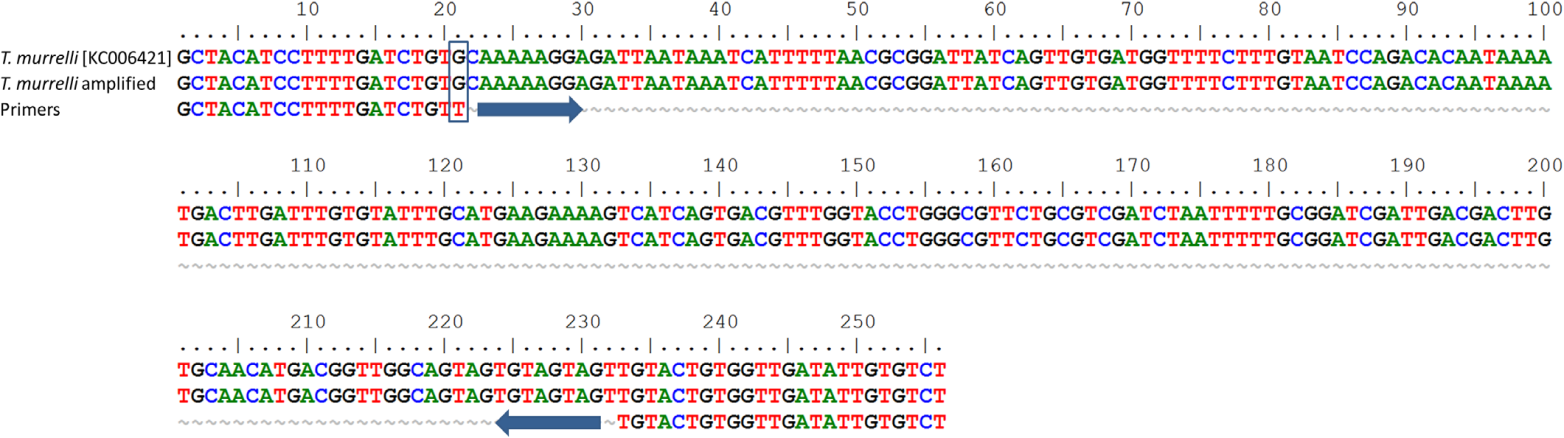
Alignment of the 256 bp fragment of ITS1 II of *Trichinella murrelli* obtained by uniplex PCR.BLAST analysis revealed 99.6% identity with different clones of *T. murrelli*, including clone 5 (Accession number KC006421).

Since the 256 bp band produced by *T. murrelli* was unexpected, a uniplex PCR was performed to identify which couple of primers allowed the amplification of the extra band. The 256 bp band amplified with primer pair II for ITS1 (Figure 2) was sequenced and identified by BLAST. The result revealed 99.6% identity with *T. murrelli* (GenBank accession number KC006421). Only one base was different and corresponded to the last base of the forward primer-annealing region (Figure 3). It follows that the complementarity of the forward primer is not 100% and this may explain the intermittent amplification of the 256 bp product. Slightly different PCR conditions may affect annealing, resulting in two- or three-band patterns.

The appearance of a third unexpected band using DNA of *T. murrelli* larvae by the multiplex PCR analysis described by Zarlenga *et al.*, (1999) [24] may be the cause of misinterpretation, leading the analyst to suppose a *T. murrelli*/*T. britovi* hybrid or cross DNA contamination of the purified DNA sample under analysis. Incorrect identification of *T. murrelli* larvae occurred in 2016 during the proficiency testing organized by the EURLP for the NRLs to identify *Trichinella* larvae at the species level. Seven (33%) of the 21 participating laboratories failed to identify *T. murrelli* by multiplex PCR due to the appearance of the unexpected band of 256 bp (Final Report PT-Tm 1/2016; www.iss.it/dinary/crlp/cont/Final_report_PT_Tm_2016.pdf).

The appearance of the extra band of 256 bp in *T. murrelli* was previously documented [18], but since this band was generated intermittently, it was not considered diagnostic of *T. murrelli* and was consequently ignored.

## Conflict of interest

The authors declare that they have no conflict of interest.
